# Identification of diborane(4) with bridging B–H–B bonds†
†Electronic supplementary information (ESI) available: Ultraviolet absorption spectrum of B_2_H_6_ dispersed in solid neon, infrared absorption lines recorded after photolysis of B_2_H_6_/Ne = 1/1000 or B_2_D_6_/Ne = 1/1000 at 122.6 nm, emission spectrum from B_2_H_6_/Ne = 1/1000 at 3 K irradiated at 122.6 nm, UV absorption spectrum of B_2_ from B_2_H_6_/Ne = 1/1000 at 3 K irradiated at 122.6 nm, temporal profiles of photolysis products of B_2_H_6_/Ne = 1/1000 at 3 K after irradiation at 122.6 nm; calculated structures, enthalpies of formation, vibrational wavenumbers and intensities for various B_2_H_*n*_ species, wavenumber/cm^–1^ and intensity/km mol^–1^ of calculated fundamental vibrational modes for various isotopic B_2_H_4_^+^ (*C*_2*v*_) and B_2_D_4_^+^ (*C*_2*v*_), wavenumber/cm^–1^ and intensity/km mol^–1^ of calculated fundamental vibrational modes and NIST data for ^11^B_2_H_6_. See DOI: 10.1039/c5sc02586a


**DOI:** 10.1039/c5sc02586a

**Published:** 2015-08-14

**Authors:** Sheng-Lung Chou, Jen-Iu Lo, Yu-Chain Peng, Meng-Yeh Lin, Hsiao-Chi Lu, Bing-Ming Cheng, J. F. Ogilvie

**Affiliations:** a National Synchrotron Radiation Research Center , No. 101, Hsin-Ann Road, Hsinchu Science Park , Hsinchu 30076 , Taiwan . Email: bmcheng@nsrrc.org.tw; b Escuela de Quimica , Universidad de Costa Rica , Ciudad Universitaria Rodrigo Facio , San Pedro de Montes de Oca , San Jose 11501-2060 , Costa Rica

## Abstract

The irradiation of diborane(6) dispersed in solid neon at 3 K with tunable far-ultraviolet light from a synchrotron yielded a set of IR absorption lines, the pattern of which implies a carrier containing two boron atoms. According to isotope effects and quantum-chemical calculations, we identified this new species as diborane(4), B_2_H_4_, possessing two bridging B–H–B bonds. Our work thus establishes a new prototype, diborane(4), for bridging B–H–B bonds in molecular structures.

## Abstract 2

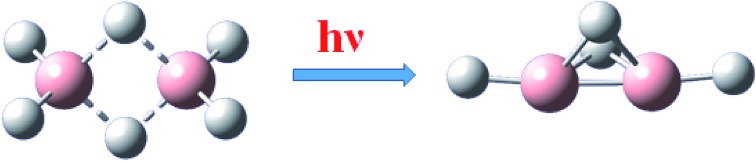
The irradiation of diborane(6) dispersed in solid neon at 3 K with far-ultraviolet light generated diborane(4), B_2_H_4_, with bridging B–H–B bonds.

## Introduction

The structure that B_2_H_6_ adopts in its electronic ground state and most stable conformation contains two bridging B–H–B bonds, and four terminal B–H bonds. Although Bauer, from experiments with the electron diffraction of gaseous samples in the laboratory of Pauling and Brockway, favoured a structure of diborane(6) with a central B–B bond analogous to that of ethane,^1^ Longuet-Higgins deduced the bridged structure for diborane(6) largely from the infrared spectra of gaseous samples, and correctly predicted the structures of other borohydrides.^2^,^3^ Subsequent measurements of diborane(6) and other boron hydrides using X-ray diffraction were consistent with such bridged structures in the solid phase;^4^ as the structures of other boron compounds B_2_F_4_ and B_2_Cl_4_ (ref. 5) in the crystalline phase differ from those of the free molecules, this crystal structure of B_2_H_6_ is not necessarily applicable to molecules in the gaseous phase. Since this work to characterize boron hydrides, other molecular species with bridging hydrogen atoms have been detected and proposed.^6^

The B–H–B linkage in these compounds is considered to be an atypical electron-deficient covalent chemical bond.^7^ According to calculations, diborane species possessing less than 6 hydrogen atoms and bridging B–H–B bonds can exist,^8^–^13^ but all possible candidates are transient species, difficult to prepare and to identify. Since the pioneering work involving absorption spectra of samples at temperatures less than 100 K,^14^,^15^ many free radicals and other unstable compounds dispersed in inert hosts have been detected.^16^–^20^ Investigations of transient boron species using the matrix-isolation technique have been reported.^21^–^25^ For instance, reactions of H_2_ with boron atoms evaporated with a pulsed laser and condensed in excess argon at 10 K yielded products such as BH, (H_2_)(BH) complex, BH_3_, (H_2_)(BH_3_) complex, HBBH and B_2_H_6_, identified from infrared absorption spectra.^23^ The irradiation of samples of B_2_H_6_ diluted in neon or argon at 4 K with light at 16.8 eV followed by X-rays and electrons at 50 eV enabled the detection of BH_2_, BO, B_2_ and HBBH through electron-paramagnetic-resonance spectra,^24^ but no neutral boron hydride species with less than six H atoms and a bridging B–H–B bond has been experimentally identified before our work.

When we irradiated diborane(6) dispersed in neon at 3 K with far-ultraviolet light, we recorded many new lines in the infrared absorption spectra and ultraviolet emission and absorption spectra. Among the infrared lines, a set with common properties of growth and decay was characteristic of a carrier containing two boron atoms, which we identified as diborane(4), B_2_H_4_, according to isotopic shifts, both from ^10^B and ^11^B and from H and D, consistent with the results of the quantum-chemical calculations of the vibrational wavenumbers. Among the several species that we detected, this species with two terminal hydrogen atoms possessing two bridging hydrogen atoms in its molecular structure becomes the simplest neutral boron hydride identified with such a structural feature.

## Results and discussion

Our experiments involved the photolysis of diborane(6) dispersed in solid neon with far-ultraviolet light from beamline BL21A2 in the National Synchrotron Radiation Research Center (NSRRC).^26^–^28^ Upon irradiation of a sample with light in the wavelength range of 120–220 nm, the intensity of all infrared absorption lines of diborane(6) in all samples at 3 K decreased uniformly and continuously, but with a rate decreasing with an increasing duration of irradiation; the extent and rate of depletion, and the particular new lines appearing after photolysis, depended on the selected wavelength.

For a sample with B_2_H_6_/Ne = 1/1000 deposited as a film at 3 K, the absorption spectrum in the range of 115–220 nm is presented in Fig. S1 of the ESI;† this spectrum was recorded on beamline BL03.^29^,^30^ The most intense feature of this ultraviolet spectrum has maximum absorption at 122.6 nm; the irradiation of solid B_2_H_6_/Ne = 1/1000 at this wavelength produced many new lines in the infrared absorption spectrum, listed in Table S1 in the ESI.†

In other experiments, we varied the molar ratio of the diborane(6) precursor to the neon dispersant from 1 : 100 to 1 : 10 000; we varied also the wavelength of the intense light, continuously tunable from the synchrotron source, and recorded the infrared absorption spectra after successive periods of 10, 30, 60, 300, 600, 1800, 1800, 3600, 3600 and 7200 s of irradiation of the sample at the selected wavelength. Of these new lines, some are directly assigned to known species on the basis of published data, including BH,^23^ BH_3_,^23^,^25^ and B_2_H_2_,^23^ as listed in Table S1;† for the other lines, the variation of their relative intensities according to the varied wavelength of excitation and curve of growth enabled their assignment to a distinct carrier, as indicated in Table 1. The emission and absorption spectra in the 200–1000 nm region recorded concurrently with photolysis showed evidence for the production of BH,^31^,^32^ BH_2_,^33^ BH_3_,^33^ B_2_,^34^ and H and B atoms^35^–^38^ (see Fig. S2 and S3 in the ESI†).

**Table 1:** Wavenumber (cm^–1^) and intensity (km mol^–1^) of the calculated fundamental vibrational modes and the experimental wavenumber (cm^–1^) of B_2_H_4_ and B_2_D_4_ in various isotopic forms

Mode (sym)	^11^B_2_H_4_			^11^B^10^BH_4_			^10^B_2_H_4_		
	Calc.^*a*^/cm^–1^ (int.)	Calc.^*b*^/cm^–1^	Exp./cm^–1^	Calc.^*a*^/cm^–1^ (int.)	Calc.^*b*^/cm^–1^	Exp./cm^–1^	Calc.^*a*^/cm^–1^ (int.)	Calc.^*b*^/cm^–1^	Exp./cm^–1^
ν_1_ (A_1_)	2738.4 (0.01)	2734.0	—	2749.7 (1.26)	2747.0	—	2758.2 (0.02)	2752.9	—
ν_2_ (A_1_)	2001.1 (25.0)	1999.6	1992.6	2001.8 (25.1)	2043.6	—	2002.8 (25.1)	2005.0	—
ν_3_ (A_1_)	1317.3 (2.85)	1321.3	1318.6	1344.9 (5.40)	1343.6	1343.0	1370.2 (2.91)	1392.5	—
ν_4_ (A_1_)	1089.3 (0.66)	1119.9	—	1092.0 (0.69)	1030.6	—	1094.2 (0.67)	1036.4	—
ν_5_ (A_1_)	710.4 (5.03)	723.7	719.1	711.4 (5.09)	724.5	720.9	712.5 (5.11)	725.6	721.9
ν_6_ (A_2_)	1195.4 (0.00)	1056.4	—	1195.5 (0.00)	1056.9	—	1195.4 (0.00)	1057.2	—
ν_7_ (A_2_)	619.6 (0.00)	548.7	—	626.1 (0.00)	554.5	—	632.7 (0.00)	560.72	—
ν_8_ (B_1_)	2002.2 (55.1)	2038.1	1996.4	2005.5 (55.3)	2003.3	1999.9	2008.9 (55.4)	2047.8	—
ν_9_ (B_1_)	773.9 (1.39)	784.4	—	774.9 (1.37)	785.1	—	776.0 (1.34)	786.3	—
ν_10_ (B_2_)	2699.8 (35.2)	2702.4	2695.7	2704.6 (34.9)	2708.0	2700.4	2711.3 (37.0)	2711.1	2707.4
ν_11_ (B_2_)	1279.3 (189.5)	1137.7	1278.1	1280.4 (187.7)	1140.2	1279.1	1283.1 (191.2)	1140.5	1281.2
ν_12_ (B_2_)	532.3 (43.9)	522.4	540.2	537.8 (45.1)	527.4	545.2	543.3 (46.4)	532.7	550.5

Mode (sym)	^11^B_2_D_4_			^11^B^10^BD_4_			^10^B_2_D_4_		
	Calc.^*a*^/cm^–1^ (int.)	Calc.^*b*^/cm^–1^	Exp./cm^–1^	Calc.^*a*^/cm^–1^ (int.)	Calc.^*b*^/cm^–1^	Exp./cm^–1^	Calc.^*a*^/cm^–1^ (int.)	Calc.^*b*^/cm^–1^	Exp./cm^–1^
ν_1_ (A_1_)	2102.8 (0.34)	2114.5	—	2123.5 (0.87)	2134.5	—	2141.7 (0.47)	2152.0	—
ν_2_ (A_1_)	1429.6 (12.9)	1470.3	1415.3	1431.0 (13.0)	1475.9	1417.9	1432.6 (13.0)	1478.7	—
ν_3_ (A_1_)	1209.6 (1.60)	1218.8	—	1227.9 (1.64)	1236.2	—	1245.1 (1.55)	1252.5	—
ν_4_ (A_1_)	801.6 (0.20)	805.8	—	804.7 (0.20)	809.0	—	807.4 (0.17)	812.0	—
ν_5_ (A_1_)	515.5 (2.82)	525.7	523.8	516.6 (2.92)	526.7	524.8	517.6 (2.87)	527.6	—
ν_6_ (A_2_)	845.6 (0.00)	785.1	—	845.6 (0.00)	785.1	—	845.6 (0.00)	784.9	—
ν_7_ (A_2_)	524.2 (0.00)	490.4	—	531.5 (0.01)	497.2	—	539.6 (0.00)	503.9	—
ν_8_ (B_1_)	1463.5 (28.7)	1510.1	1477.5	1468.1 (28.9)	1513.2	1481.3	1472.8 (29.0)	1522.2	—
ν_9_ (B_1_)	561.0 (0.49)	571.2	—	562.5 (0.46)	572.0	—	563.4 (0.46)	573.6	—
ν_10_ (B_2_)	1990.8 (27.3)	2015.8	—	1997.8 (27.9)	2023.1	—	2006.8 (29.4)	2033.9	—
ν_11_ (B_2_)	929.6 (103.2)	859.4	921.6	931.5 (103.9)	861.0	923.6	933.9 (104.8)	862.8	926.7
ν_12_ (B_2_)	447.7 (34.3)	445.2	457.3	453.7 (35.4)	451.2	463.4	460.1 (36.7)	457.8	—

^*a*^These wavenumbers (cm^–1^), scaled by 0.967 (intensities (km mol^–1^) within parentheses), are calculated with a harmonic approximation.

^*b*^These wavenumbers (cm^–1^) are calculated with an anharmonic approximation.

From precursor B_2_H_6_, one group of new infrared absorption numbers in seven distinct sets implies the presence of a carrier containing at least five atoms. Our sample of diborane contains boron in its natural abundance, so with two isotopic variants, ^10^B and ^11^B, with a ^11^B : ^10^B ratio about 4. We count as a set two or three closely spaced lines that exhibit the same intensity behaviour and likely arise from boron isotopes within the same carrier. The pattern of absorption at particular locations, which hence comprises triplets of varied intervals, indicates that the carrier contains two boron atoms, yielding three lines due to molecules containing ^11^B_2_, ^11^B^10^B and ^10^B_2_, in order of increasing wavenumber and decreasing intensity (∼16 : 8 : 1) within a particular pattern.

**Fig. 1:**
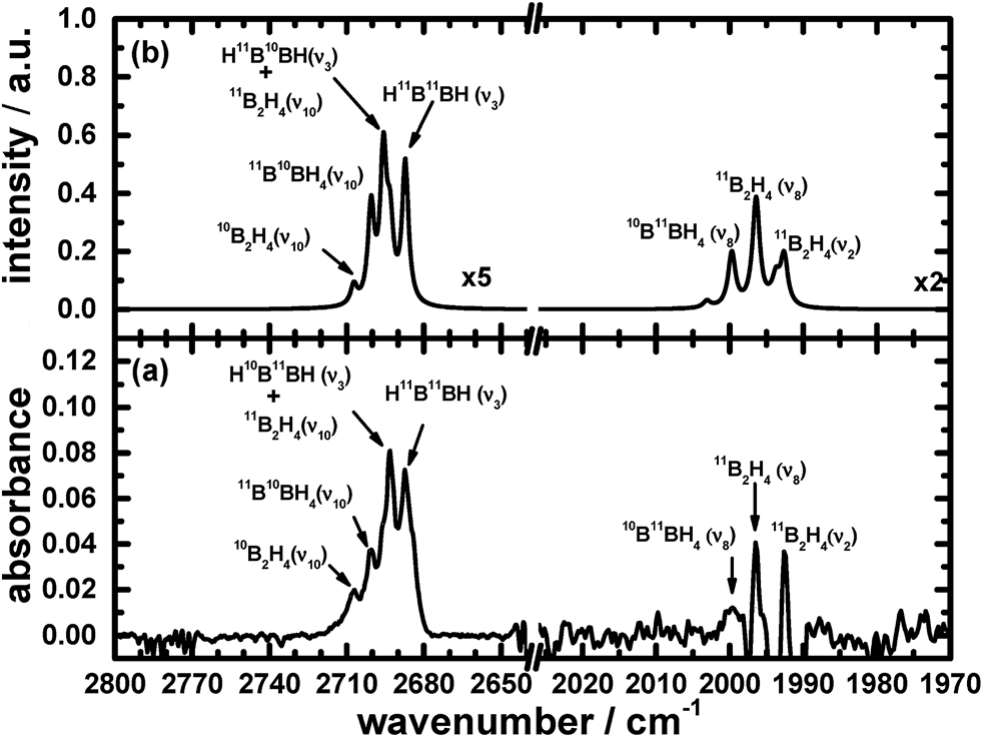
Infrared absorption of B_2_H_4_ in the ν_2_, ν_8_ and ν_10_ modes (a) from the photolysis of B_2_H_6_/Ne = 1/1000 at 3 K upon excitation at 122.6 nm (resolution 0.5 cm^–1^ from 1000 coadded interferograms), and (b) from simulations. Some assignments are indicated.

**Fig. 2:**
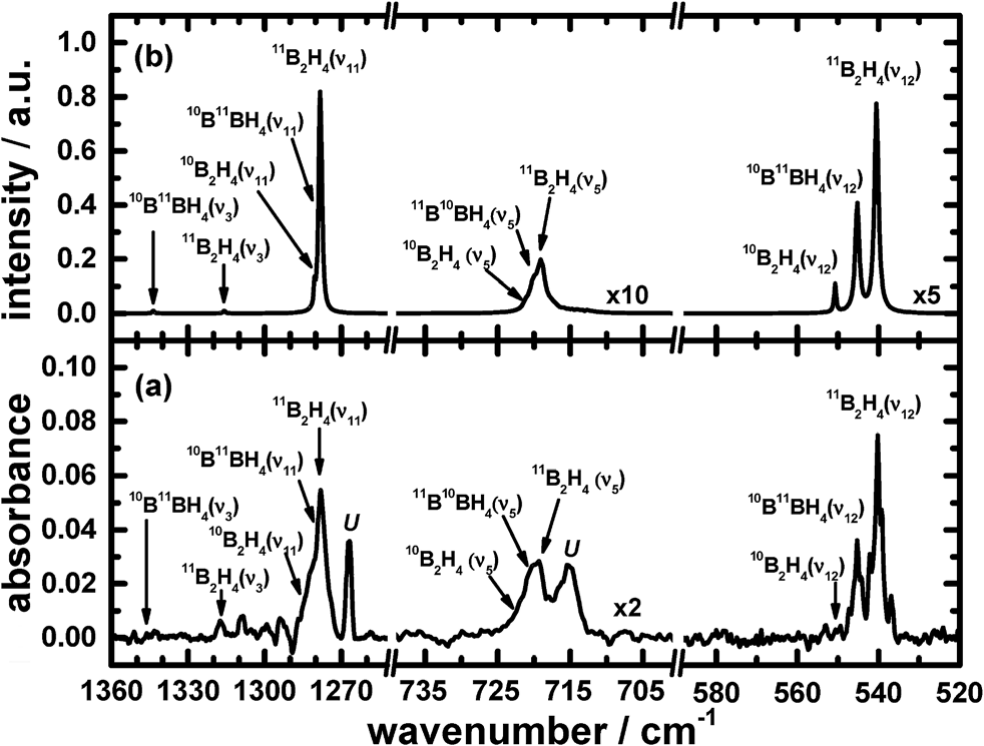
Infrared absorption of B_2_H_4_ in the ν_3_, ν_5_, ν_11_ and ν_12_ modes (a) from the photolysis of B_2_H_6_/Ne = 1/1000 at 3 K upon excitation at 122.6 nm (resolution 0.5 cm^–1^ from 1000 coadded interferograms), and (b) from simulations. Some assignments are indicated; “*U*” signifies an unidentified carrier.

The remaining atoms within that carrier must hence number at least three hydrogen atomic centres. Among these lines, a triplet at 2693.1, 2700.4 and 2707.4 cm^–1^ indicates the presence of terminal BH moieties, and lines at 1992.6, 1996.4 and 1999.9 cm^–1^ indicate bridging BH moieties, similarly to the spectrum of precursor B_2_H_6_. Based on the lack of similarity of the features in this set to the calculated wavenumbers published for B_2_H_3_,^39^ or from our analogous calculations (see ESI†), we exclude the possibility of B_2_H_3_ being the carrier of this spectrum. Fig. 1 and 2 show the infrared spectra after photolysis in selected regions exhibiting the features attributed to B_2_H_4_.

**Fig. 3:**
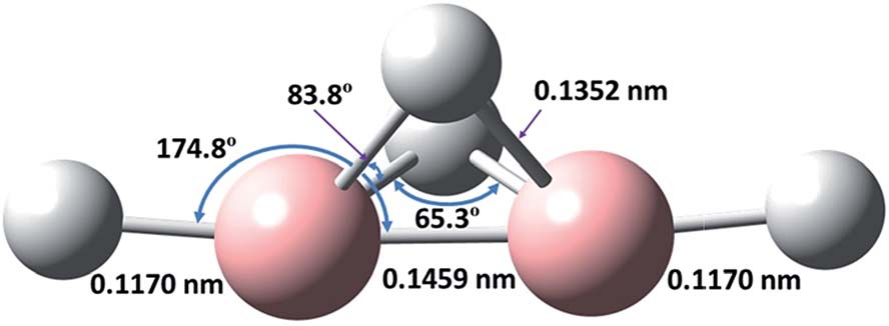
Calculated structure of B_2_H_4_ (*C*_2*v*_).

To provide additional evidence for our assignments, we undertook new quantum-chemical calculations of the harmonic and anharmonic vibrational motions of diborane(4) in its fundamental modes with the Gaussian 09 program, B3LYP method and 6-311++G** (B3LYP/6-311++G**) basis set, as listed in Table 1. The geometry of diborane(4) was analyzed using the natural-bond-orbital (NBO) method. Fig. 3 displays the calculated structural parameters of diborane(4) with point group *C*_2*v*_ symmetry; as indicated, the structure contains two bridging B–H–B bonds and likely a B–B bond.

**Fig. 4:**
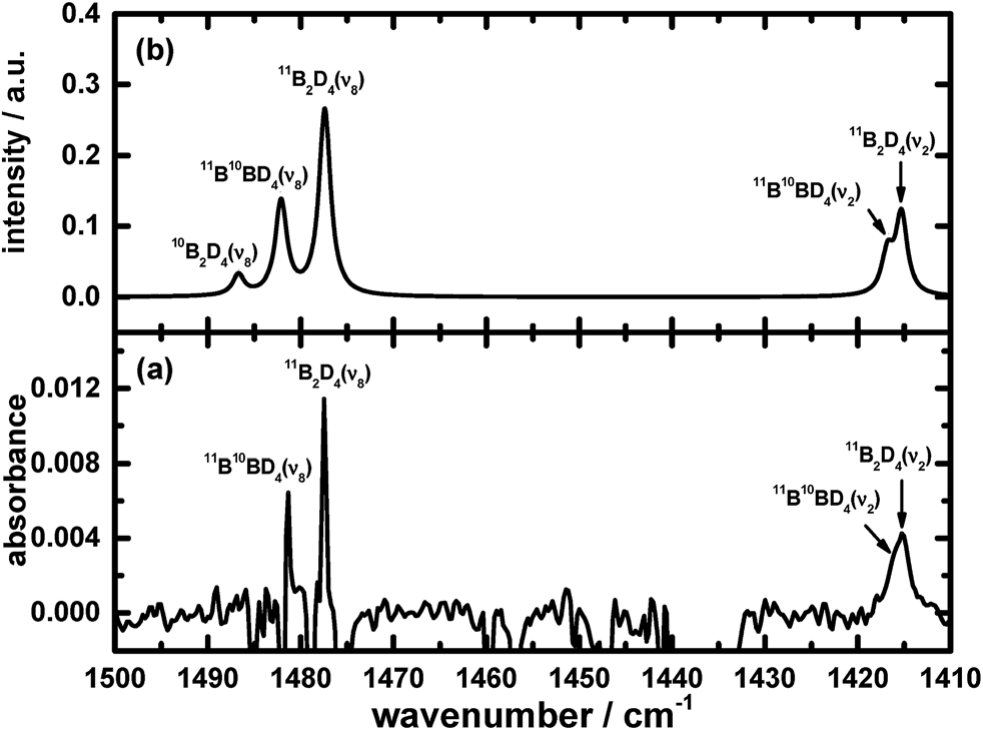
Infrared absorption spectra of B_2_D_4_ in the ν_2_ and ν_8_ modes (a) from the photolysis of B_2_D_6_/Ne = 1/1000 at 3 K upon excitation at 122.6 nm (resolution 0.5 cm^–1^ from 1000 coadded interferograms), and (b) from simulations. Some assignments are indicated.

**Fig. 5:**
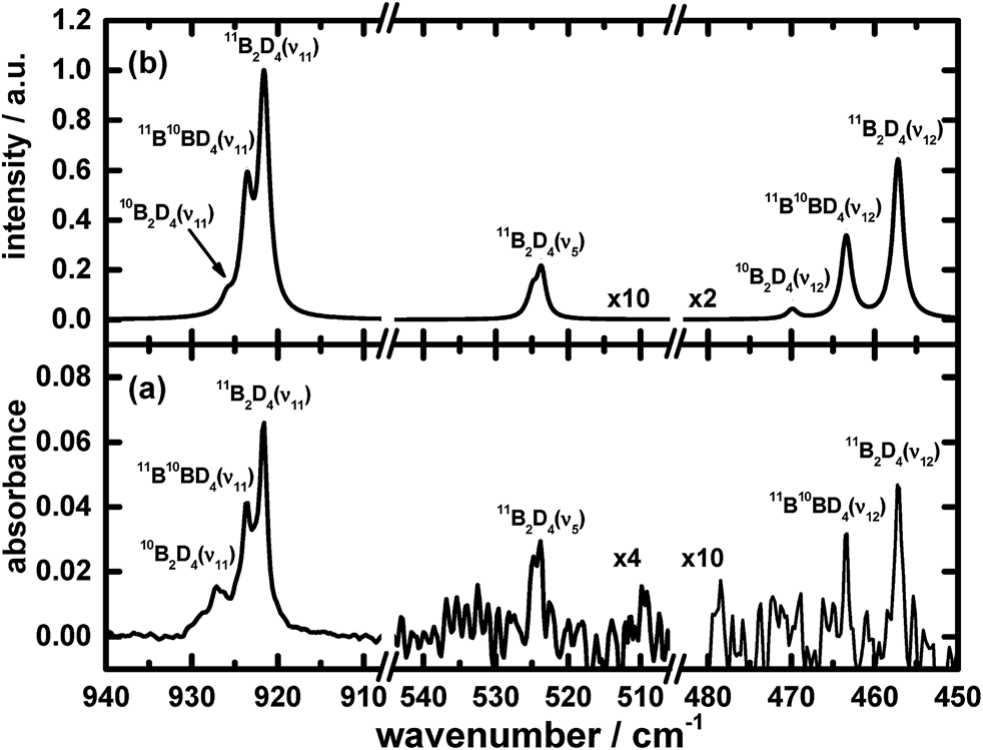
Infrared absorption spectra of B_2_D_4_ in the ν_5_, ν_11_ and ν_12_ modes (a) from the photolysis of B_2_D_6_/Ne = 1/1000 at 3 K upon excitation at 122.6 nm (resolution 0.5 cm^–1^ from 1000 coadded interferograms), and (b) from simulations. Some assignments are indicated.

The results of these calculations for B_2_H_4_ provide the wavenumbers for vibrational modes, from both the scaled harmonic and anharmonic calculations, in sets consistent with the selected new lines in the spectrum. The correspondingly calculated wavenumbers for B_2_D_4_ from precursor B_2_D_6_ are likewise consistent with the absorption lines for five vibrational modes of B_2_D_4_ with the same structure, as presented in Table 1, Fig. 4 and 5. For some vibrational modes, the features were too weak for observation of the entire triplet due to the boron isotopes; the more dominant carriers containing ^11^B_2_ and ^11^B^10^B, or just ^11^B_2_, were detected in such cases. The symmetry classes of the vibrations in the fundamental modes specified in Table 1 apply formally to ^11^B_2_H_4_ and ^10^B_2_H_4_, and their fully deuterated counterparts, belonging to point group *C*_2*v*_; for ^11^B^10^BH_4_ and ^11^B^10^BD_4_, the pertinent point group is *C*_s_ and the vibrational modes belong to the symmetry classes A′ and A′′, but the order in Table 1 is based on the correlation with the modes corresponding to point group *C*_2*v*_.

Fig. 1, 2, 4 and 5 not only show the experimental spectra in selected regions but also the simulations of pertinent regions based on the calculated anharmonic wavenumbers and the calculated harmonic intensities presented in Table 1. The intensities are unavailable for the anharmonic calculations. The wavenumbers of the features in the simulated plots are slightly shifted from their values, as stated in Table 1, to correlate directly with the experimental features.

Among the many new lines that appeared in the infrared absorption spectra, it is important to emphasize that the lines listed in Table 1 that are attributed to the new species diborane(4) as a result of the photolysis of precursor B_2_H_6_ are distinguished from the other lines due to experimental evidence. For instance, the temporal dependence of their intensity has a common rate of increase during the photolysis, as shown in Fig. S4 in the ESI,† distinct from the temporal dependence of the other lines. The rate of production of the lines attributed to diborane(4) matches the rate of depletion of the respective precursor diborane(6), indicating that diborane(4) is likely a primary photochemical product. Also, during an annealing operation in which the temperature of a sample of neon containing remaining diborane(6) and various products after the photolysis or secondary reactions was raised to 8 or 9 K for 10 to 20 min and then recooled to 3 K, the intensities of the lines associated with diborane(4) in the subsequent spectra were slightly enhanced, whereas the intensities of the other lines decreased. Furthermore, the threshold of the production of lines due to diborane(4) was about 180 nm, whereas the other product lines appeared upon photolysis at 200 or 220 nm, for instance the lines at 2686.1 and 2693.1 cm^–1^ assigned to B_2_H_2_.

A crucial aspect of this experimental discovery of diborane(4) is the nature of the molecular conformation or structure. For diborane(6), the original idea was that its structure was analogous to that of ethane; the results of the first experiments on electron diffraction were interpreted as being consistent with that structure,^1^ whereas further experiments of the same type directly confirmed the doubly bridged structure.^40^ The standard enthalpy of formation of gaseous diborane in its stable bridged structure, Δ*H*_f_, is endothermic by 41 kJ mol^–1^; no value is reported for the hypothetical unbridged structure because calculations fail to indicate such a stable conformation: from an initial structure analogous to that of ethane, the energy decreases monotonically toward dissociation into two stable, planar BH_3_ molecules. Our calculations for Δ*H*_f_ of B_2_H_4_ in the doubly bridged structure (207 kJ mol^–1^) and in the unbridged structure (220 kJ mol^–1^) differ by only 13 kJ mol^–1^, which might be comparable with an uncertainty in the calculations due to the nature of the basis sets or with various approximations; in any case, the fact that the doubly bridged structure is calculated to be slightly more stable is consistent with our experimental results, and lends confidence to the vibrational wavenumbers calculated on that basis (see ESI†). The latter values, for either the scaled harmonic wavenumbers or the anharmonic wavenumbers, exhibit significant differences with the measured wavenumbers of the selected lines, even though the pattern of the wavenumbers is similar; in most cases these differences are larger than the expected shifts between the values for diborane(4) molecules dispersed in solid neon and the values expected for the respective free molecules in the gaseous phase; the molecules in the solid phase are in an uncertain and variable environment depending on the neighbouring atoms or molecules. These differences between the observed and calculated wavenumbers are typical of such calculations, and by no means invalidate the conclusion on the general similarities of the patterns of the corresponding experimental and calculated values for the various vibrational modes.

Many calculations of neutral and ionic B_2_H_4_ have appeared in the literature;^8^–^13^ among these results, neutral and ionic diborane(4) species having structures containing bridging B–H–B bonds have been proposed. The B_2_H_4_^+^ cation was detected after photoionization in a mass spectrometer,^41^ but neutral B_2_H_4_ has not previously been directly observed experimentally; our vibrational wavenumbers calculated for B_2_H_4_ fit our recorded spectra better than those calculated for B_2_H_4_^+^ (see ESI†), and there is no evidence for the production of molecular ions under our experimental conditions.

Shoji *et al.* reported the isolation of a terminally disubstituted diborane(4) compound, stabilized by bulky groups, such as 1,1,3,3,5,5,7,7-octaethyl-*s*-hydrindacen-4-yl (Eind) groups.^42^ The frame of the molecular structure of this substituted diborane(4) was proposed to be doubly hydrogen-bridged and of a butterfly shape, similar to that seen in Fig. 3; this structure was determined by X-ray crystallography and NMR spectra. The crystal is stable near 295 K for more than one year in the absence of air. The bulky Eind groups might well stabilize the structure of the EindB(μ-H)_2_BEind (μ-H indicates the bridging H atom) in a particular form, whereas diborane(4) lacks those spatial constraints and can exhibit the natural structure of a boron hydride. Our observation and identification of neutral B_2_H_4_ from the photolysis of diborane(6) dispersed in solid neon near 3 K at 122.6 nm hence make it a new prototype for a simple species with bridging B–H–B bonds.

## Experiments

Undulator beamline 21A2 attached to the 1.5 GeV storage ring at the Taiwan Light Source (TLS) in the National Synchrotron Radiation Research Center (NSRRC) provided pseudo-continuous far-ultraviolet light with a photon flux ∼10^16^ photons s^–1^ (2% bandwidth). Absorption by Ar (pressure 1.33 kPa) and a filter – crystalline LiF for 121.6 nm, CaF_2_ for 130, 140 and 155 nm, silica (Suprasil) for 165 and 175 nm, and quartz for 185, 190 and 200 nm – suppressed the harmonics from the undulator.

The apparatus for this work is described elsewhere.^27^–^29^ A gaseous sample, mixed well, containing diborane(6) and neon in great excess was deposited on a KBr window cooled to 3 K in a closed-cycle cryostat (Janis RDK-415), which was evacuated to less than 1.3 × 10^–6^ Pa with a turbomolecular pump backed with a scroll pump. This cryostat was situated on the plate of a differential rotary-seal stage of which the rotatable angle is 360°. The KBr window can thus be rotated freely to face the deposition, photolysis or detection ports.

The infrared absorption spectra were recorded in transmission through a sample at various stages of the experiments with an interferometric spectrometer (Bomem, DA8, KBr beamsplitter and HgCdTe detector cooled to 77 K) from 450 to 4000 cm^–1^ with resolution 0.5 cm^–1^. Ultraviolet and visible absorption and emission were analyzed with a monochromator (iHR320). The dispersed ultraviolet and visible light was detected with photon detectors of two types, either a photomultiplier tube (Hamamatsu R928, photon-counting mode) or a charge-coupled detector (1024 × 256 pixels, Horiba Symphony II, image mode). The entrance slit at width 0.5 mm produced a resolution about 0.2 nm with CCD detection.

Before and after the irradiation of the sample for cumulative periods from 0.5 to 12 h in a programmed sequence, we recorded, at each stage of the experiment, the infrared absorption, ultraviolet and visible absorption and emission spectra. The difference spectra, defined as the absorbance curve after irradiation of a sample with far-ultraviolet light for some period minus the absorbance curve before irradiation, emphasized the variation of the chemical composition resulting from the photolysis.

Precursor B_2_H_6_ or B_2_D_6_ (Voltaix, chemical purities B_2_H_6_ 99.99% and B_2_D_6_ 99.8%) was received as 10% in He.

## Conclusion

The irradiation of diborane(6) dispersed in solid neon at 3 K with tunable far-ultraviolet light from a synchrotron yielded infrared absorption lines in a set that had similar conditions of growth and decay and that indicated a carrier containing two boron atoms. According to isotope effects involving both boron – ^10^B, ^11^B – and hydrogen – H, D – the new species was assigned as diborane(4), *i.e.* B_2_H_4_, possessing two bridging hydrogen atomic centres between the two boron centres, consistent with the results of quantum-chemical calculations of the vibrational wavenumbers. Our work thus established a new prototype, diborane(4), for bridging B–H–B bonds in a molecular structure, a derivative of which, with bulky terminal substituents, has already been characterized.^42^

## Supplementary Material

Supplementary information
