# Supplementary material for: Identification of diborane(4) with bridging B–H–B bonds
Source: Chem Sci. 2015 Aug 14;6(12):6872–7. doi: 10.1039/c5sc02586a (PMC5951111; doi:10.1039/c5sc02586a)
Supplement: Supplementary file 1 [file SC-006-C5SC02586A-s001.pdf]

Electronic Supplementary Material (ESI) for Chemical Science.
This journal is © The Royal Society of Chemistry 2015

Supplementary Information

Identification of Diborane(4) with Bridging B-H-B Bonds

Sheng-Lung Chou,[1] Jen-Iu Lo,[1] Yu-Chain Peng,[1] Meng-Yeh Lin,[1] Hsiao-Chi Lu,[1]

Bing-Ming Cheng[1]* and J. F. Ogilvie[2]

[1]*National Synchrotron Radiation Research Center, 101 Hsin-Ann Road, Hsinchu Science Park, Hsinchu 30076, Taiwan*
[2]*Escuela de Quimica y CELEQ, Universidad de Costa Rica, Ciudad Universitaria Rodrigo Facio, San Pedro de Montes de Oca, San Jose 11501-2060, Costa Rica*

*To whom correspondence should be addressed. e-mail: bmcheng@nsrrc.org.tw

This supplementary information describes ultraviolet absorption of B_2H_6 dispersed in solid neon in section 1, infrared absorption lines recorded after photolysis of B_2H_6/Ne=1/1000 or B_2D_6/Ne=1/1000 at 122.6 nm in section 2, the emission spectrum from B_2H_6/Ne=1/1000 irradiated with 122.6 nm at 3 K in section 3, the UV absorption spectrum of B_2 from B_2H_6/Ne=1/1000 irradiated with 122.6 nm at 3 K in section 4, the temporal profiles of photolysis of B_2H_6/Ne=1/1000 upon 122.6 nm at 3 K in section 5, the calculated structures, enthalpies of formation, vibrational wavenumbers and intensities for various species B_2H_n in section 6, wavenumber/cm^{-1} and intensity/km mol^{-1} of calculated fundamental vibrational modes for various isotopic $B_2H_4^+$ (C_{2v}) and $B_2D_4^+$ (C_{2v}) in section 7, and wavenumber/cm^{-1} and intensity/km mol^{-1} of calculated fundamental vibrational modes and NIST data for $^{11}B_2H_6$ in section 8.

1. Ultraviolet absorption of B_2H_6 dispersed in solid neon

In this work, we irradiated samples of B_2H_6 dispersed in solid neon with tunable ultraviolet light selected from a synchrotron source. For photolytic dissociation, it is important to know the form and the extent of the absorption of precursor molecules involved in the radiative excitation. For this purpose, a gaseous mixture of B_2H_6 with neon in proportion 1:1000 was deposited (flow rate 0.17 sccs, period 1 h) on a LiF crystal maintained at 3 K with a refrigerator system (Janis RDK-415); we subsequently measured the absorption spectrum with a double-beam apparatus coupled to beamline BL03 at National Synchrotron Radiation Research Center.[1,2] Figure S1 shows the far UV absorption spectrum of diborane(6) dispersed in neon with molar ratio 1/1000 at 3 K in the spectral region between 115 nm and 220 nm at spectral resolution 0.2 nm; the thickness of the deposited sample was about 8-10 µm. An inspection of the total absorption profile reveals that the absorption of diborane(6) dispersed in neon contains three sections, a threshold region begins 205 to 169 nm, a weak absorption region from 169 to 135 nm, and a dominant absorption in region 135-115 nm with maximum at 122.6 nm. A detailed assignment of the transitions will be reported elsewhere.[3]

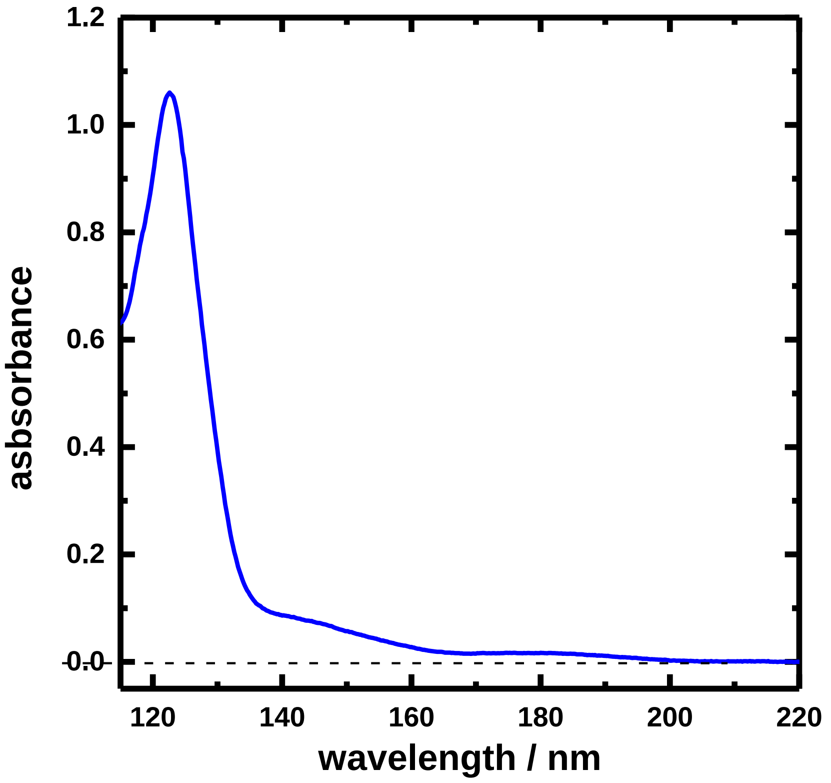

Figure S1. Absorption spectrum (resolution 0.2 nm, step 0.2 nm) of B_2H_6 dispersed in neon (1/1000) at 3 K in wavelength range 115-220 nm.

References

1. H.-C. Lu, H.-K. Chen, B.-M. Cheng, Y.-P. Kuo, J. F. Ogilvie, *J. Phys. B: At. Mol. Opt. Phys.* **38**, 3693 (2005)

2. H.-C. Lu, H.-K. Chen, B.-M. Cheng, J. F. Ogilvie, *Spectrochim. Acta B Mol. Biomol. Spectrosc.* **71**, 1485 (2008).

3. Y.-C. Peng, S.-L. Chou, J.-I. Lo, M.-Y. Lin, H.-C. Lu, B.-M. Cheng, J. F. Ogilvie, *to be submitted* (2015).

2. **Infrared absorption lines recorded after photolysis of B_2H_6/Ne=1/1000 or B_2D_6/Ne=1/1000 at 122.6 nm**

Table S1 Infrared absorption lines recorded after photolysis of B_2H_6/Ne=1/1000 or B_2D_6/Ne=1/1000 at 122.6 nm

wavenumber/cm^{-1}		species	Ref.
B_2H_6	B_2D_6		
2264.5, 2279.7	1688.5	BH	1
1141.2, 1152.3	889.9, 904.9	BH_3	1,2
2686.1, 2693.1	-	B_2H_2	1
540.2, 545.2, 550.5, 719.1, 720.9, 1278.1, 1279.1, 1281.2, 1318.6, 1343.0, 1992.6, 1999.9, 2696.0, 2700.4, 2708.3	457.3, 463.4, 523.8, 524.8, 921.6, 923.6, 926.7, 1415.3, 1417.9, 1477.5, 1481.3	B_2H_4	
536.9, 715.4, 742.5, 783.8, 835.3, 1067.4, 1209.2, 1266.8, 1294.0, 1308.2, 2315.2, 2321.7, 2391.3, 3783.4	592.8, 624.3, 791.2, 804.1, 807.1, 820.3, 823.2, 884.4, 1631.4, 1793.8, 1797.1, 1800.1, 1802.9	unidentified	

References

1. T. J. Tague, L. Andrews, *J. Am. Chem. Soc.* **116,** 4970-4976 (1994).
2. L. Andrews, X. Wang, *J. Am. Chem. Soc.* **124,** 7280-7281 (2002).

3. **Emission spectrum from B_2H_6/Ne=1/1000 at 3 K irradiated with 122.6 nm**

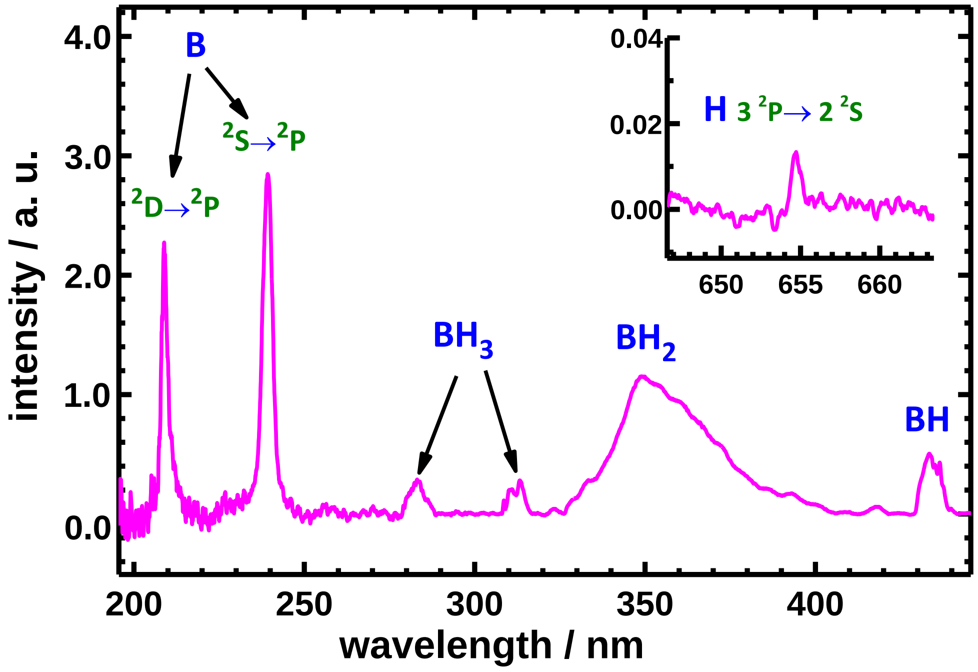

Figure S2. Emission spectrum after photolysis of B_2H_6/Ne=1/1000 at 3 K irradiated at 122.6 nm. Emission lines of products H,[1-3] B,[4] BH,[5,6] BH_2,[7] and BH_3 are identified and marked.

References

1. S. A. Mitchell, *Astrophys. J.* **105**, 1 (1947).
2. T. W. Hänsch, M. H. Nayfeh, S. A. Lee, S. M. Curry, I. S. Shahin, *Phys. Rev. Lett.* **32**, 1336–1340 (1974)..
3. P. Zhao, W. Lichten, H. P. Layer, J. C. Bergquist, *Phys. Rev. A*, **34**, 5138–5141 (1986).
4. “NIST Atomic Spectra Database,” *NIST Atomic Spectra Database*. [Online]. Available: http://www.nist.gov/pml/data/asd.cfm.
5. J. W. C. Johns, F. A. Grimm, R. F. Porter, *J. Mol. Spectr.* **22**, 435–451 (1967).
6. W. T. M. L. Fernando, P. F. Bernath, *J. Mol. Spectr*. **145**, 392–402 (1991).
7. J. A. Harrison, R. F. Meads, L. F. Phillips, *Chem. Phys. Lett.* **148**, 125–129 (1988).

4. **UV absorption spectrum of B_2 from B_2H_6/Ne=1/1000 at 3 K irradiated at 122.6 nm**

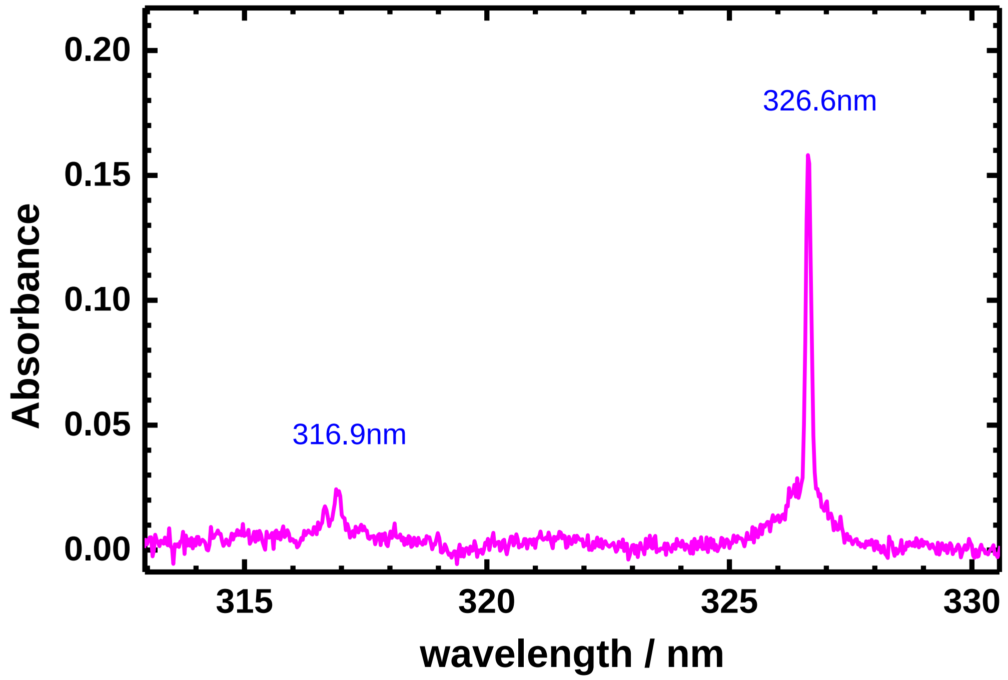

Figure S3. UV absorption spectrum of B_2 from photolysis of B_2H_6/Ne=1/1000 at 3 K at 122.6 nm. Absorption lines at 326.6 and 316.9 nm of B_2 correspond to transitions from X→A(0,0) and A(0,1),[1,2] respectively.

References

1. R. W. Nicholls, P. A. Fraser, W. R. Jarmain, *Combustion and Flame* **3**, 13–38 (1959).

2. "NIST Chemistry WebBook," *NIST Chemistry WebBook*: http://webbook.nist.gov/chemistry/.

5. **Temporal profiles of photolysis at 122.6 nm of B_2H_6/Ne=1/1000 at 3 K**

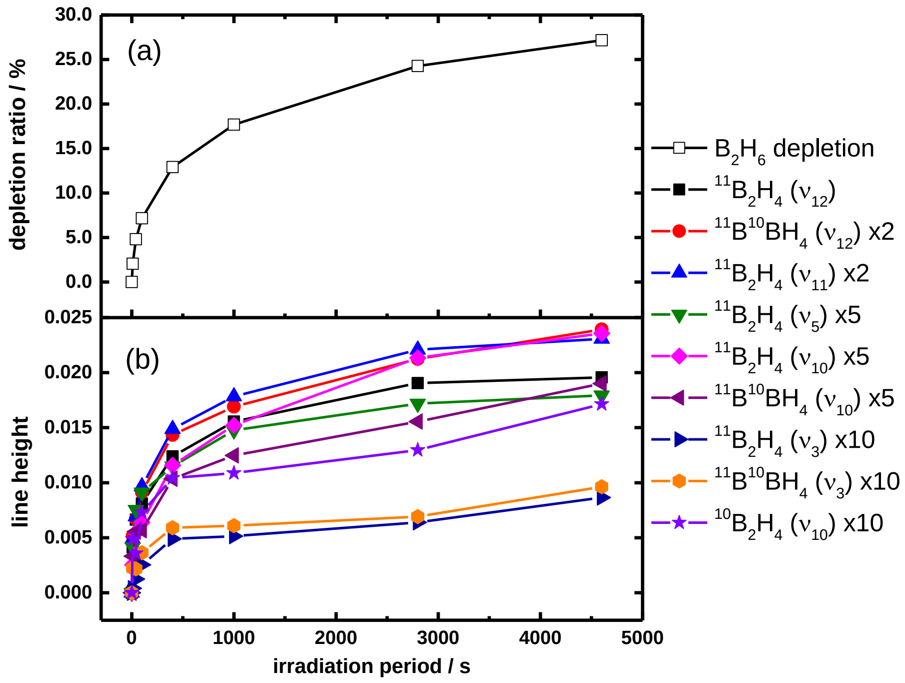

Figure S4. For B_2H_6/Ne=1/1000 at 3 K irradiated at 122.6 nm, (a) temporal profile of depletion ratio for B_2H_6; (b) temporal profiles of formation of lines attributed to B_2H_4 in various vibrational modes.

6. Calculated structures, enthalpies of formation, vibrational wavenumbers and intensities for various species B_2H_n

6(a) B_2H ($C_{\infty v}$), ΔH_f = 882.5 kJ mol^{-1}

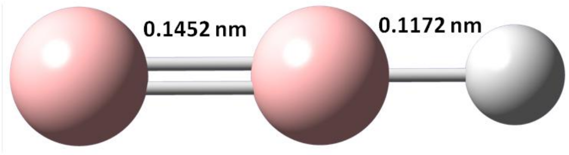

	$^{11}B_2H$	$^{10}B^{11}BH$	$^{11}B^{10}BH$	$^{11}B_2D$
mode (symmetry)	ν/cm^{-1} (int.)^a	ν /cm^{-1} (int.)^a	ν /cm^{-1} (int.)^a	ν /cm^{-1} (int.)^a
ν_1 (Σ)	2712.0 (0.8)	2712.3 (0.9)	2727.2 (1.2)	2044.5 (5.7)
ν_2 (Σ)	1316.8 (39.3)	1350.4 (41.3)	1343.0 (40.8)	1262.2 (34.1)
ν_3 (Π)	705.0 (181.1)	706.5 (182.9)	712.6 (186.9)	559.9 (129.2)

^{a}Enthalpy of formation and vibrational wavenumbers, scaled by 0.967, calculated with program Gaussian 09, B3LYP method and basis set 6-311++G** (B3LYP/6-311++G**); calculated intensities have unit km mol^{-1}.

6(b) B_2H_2 ($D_{\infty h}$), ΔH_f = 512.9 kJ mol^{-1}

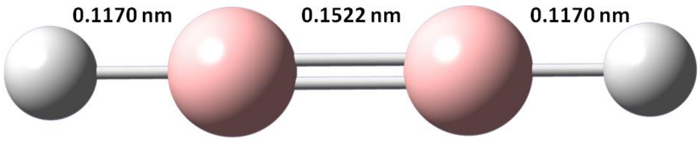

	$^{11}B_2H_2$	$^{10}B^{11}BH_2$	$^{11}B_2D_2$
mode (symmetry)	ν /cm^{-1} (int.)^a	ν / cm^{-1} (int.)^a	ν / cm^{-1} (int.)^a
ν_1 (Σ_g)	2747.2 (0.0)	2758.3 (0.6)	2091.6 (0.0)
ν_2 (Σ_g)	1194.0 (0.0)	1219.4 (0.0)	1109.8 (0.0)
ν_3 (Σ_u)	2710.1 (19.8)	2715.2 (19.2)	1996.3 (10.7)
ν_4 (Π_g)	392.3 (0.0)	403.1 (0.0)	330.9 (0.0)
ν_5 (Π_u)	568.3 (45.7)	573.1 (46.1)	421.2 (24.9)

^{a}Enthalpy of formation and vibrational wavenumbers, scaled by 0.967, calculated with program Gaussian 09, B3LYP method and basis set 6-311++G** (B3LYP/6-311++G**); calculated intensities have unit km mol^{-1}.

6(c) B_2H_3 (C_{2v}), ΔH_f = 327.0 kJ mol^{-1}

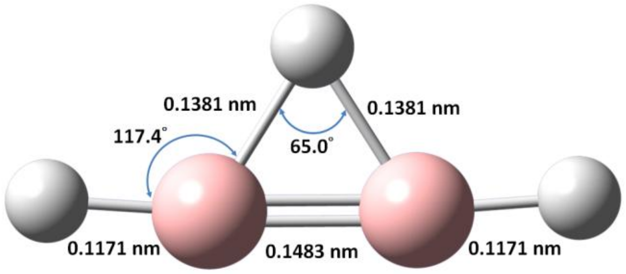

	$^{11}B_2H_3$	$^{10}B^{11}BH_3$	$^{11}B_2D_3$
mode (symmetry)	ν / cm^{-1} (int.)[a]	ν / cm^{-1} (int.)[a]	ν / cm^{-1} (int.)[a]
ν_1 (A_1)	2738.8 (0.0)	2749.5 (1.0)	2095.9 (0.1)
ν_2 (A_1)	1865.2 (102.6)	1867.9 (102.9)	1357 (53.9)
ν_3 (A_1)	1270.4 (0.6)	1297 (0.7)	1173.2 (0.2)
ν_4 (A_1)	751.5 (0.9)	752.7 (0.9)	547.6 (0.6)
ν_5 (A_2)	582.4 (0.0)	585.2 (0.3)	483.6 (0.0)
ν_6 (B_1)	586.3 (0.5)	590.0 (0.2)	429.0 (0.1)
ν_7 (B_2)	2700.4 (30.0)	2704.9 (29.5)	1989.3 (19.6)
ν_8 (B_2)	1132.9 (80.8)	1133.7 (80.4)	813.1 (37.2)
ν_9 (B_2)	458.0 (49.1)	462.7 (50.5)	385.5 (39.1)

[a]Enthalpy of formation and vibrational wavenumbers, scaled by 0.967, calculated with program Gaussian 09, B3LYP method and basis set 6-311++G** (B3LYP/6-311++G**); calculated intensities have unit km mol^{-1}.

6(d) B_2H_3 (C_{2v}), ΔH_f = 324.0 kJ mol^{-1}

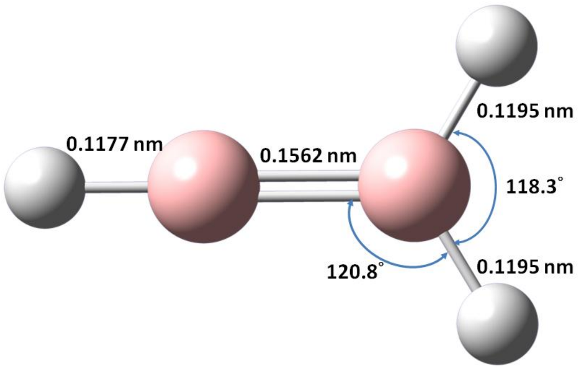

	$^{11}B_2H_3$	$^{10}B^{11}BH_3$	$^{11}B^{10}BH_3$	$^{11}B_2D_3$
mode (symmetry)	ν / cm^{-1} (int.)[a]	ν / cm^{-1} (int.)[a]	ν / cm^{-1} (int.)[a]	ν / cm^{-1} (int.)[a]
ν_1 (A_1)	2684.2 (25.7)	2697.9 (26.2)	2684.5 (25.4)	2005.3 (13.9)
ν_2 (A_1)	2470.8 (19.2)	2470.8 (19.2)	2477.2 (19.3)	1796.6 (10.6)
ν_3 (A_1)	1116.2 (1.1)	1124.8 (1.1)	1138.4 (1.2)	1016.3 (1.0)
ν_4 (A_1)	973.9 (0.1)	988 (0.1)	978.5 (0.1)	737.8 (0.0)
ν_5 (B_1)	760.8 (5.4)	761.7 (5.5)	770.6 (5.4)	612.1 (2.6)
ν_6 (B_1)	514.1 (2.7)	518.9 (2.5)	514.3 (2.6)	395.2 (0.6)
ν_7 (B_2)	2527.9 (50.9)	2527.9 (51)	2541.6 (50.3)	1884.3 (22.5)
ν_8 (B_2)	867.6 (18.1)	878.2 (18.9)	871.7 (18.6)	708.7 (16.3)
ν_9 (B_2)	309.2 (0.1)	309.2 (0.1)	310.5 (0.1)	226.8 (0.1)

[a]Enthalpy of formation and vibrational wavenumbers, scaled by 0.967, calculated with program Gaussian 09, B3LYP method and basis set 6-311++G** (B3LYP/6-311++G**); calculated intensities have unit km mol^{-1}.

6(e) B_2H_4 (C_{2v}), ΔH_f = 206.8 kJ mol^{-1}

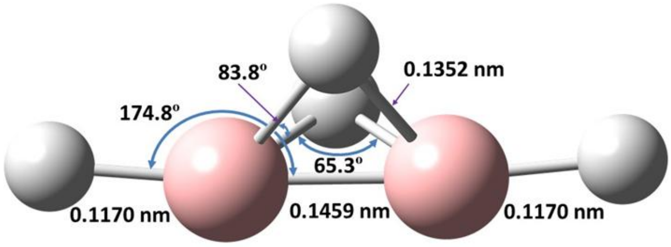

	$^{11}B_2H_4$	$^{10}B^{11}BH_4$	$^{11}B_2D_4$
mode (symmetry)	ν / cm^{-1} (int.)[a]	ν / cm^{-1} (int.)[a]	ν / cm^{-1} (int.)[a]
ν_1 (A_1)	2739.1 (0.01)	2750.2 (1.26)	2103.4 (0.34)
ν_2 (A_1)	2001.1 (25.02)	2002.1 (25.05)	1429.7 (12.88)
ν_3 (A_1)	1317.8 (2.85)	1345.4 (5.40)	1210.1 (1.60)
ν_4 (A_1)	1089.3 (0.66)	1091.8 (0.68)	801.6 (0.20)
ν_5 (A_1)	710.1 (5.03)	711.2 (5.09)	515.3 (2.83)
ν_6 (A_2)	1195.9 (0.00)	1195.9 (0.00)	845.9 (0.00)
ν_7 (A_2)	619.7 (0.00)	626.3 (0.00)	524.3 (0.00)
ν_8 (B_1)	2002.6 (55.02)	2005.9 (55.14)	1463.8 (28.68)
ν_9 (B_1)	773.7 (1.40)	774.7 (1.37)	560.8 (0.49)
ν_{10} (B_2)	2700.5 (35.19)	2705.0 (34.86)	1991.3 (27.29)
ν_{11} (B_2)	1279.6 (189.35)	1280.7 (187.67)	929.8 (103.08)
ν_{12} (B_2)	532.3 (43.80)	537.6 (45.03)	447.3 (34.21)

[a]Enthalpy of formation and vibrational wavenumbers, scaled by 0.967, calculated with program Gaussian 09, B3LYP method and basis set 6-311++G** (B3LYP/6-311++G**); calculated intensities have unit km mol^{-1}.

6(f) B_2H_4 (D_{2d}), ΔH_f = 219.8 kJ mol^{-1}

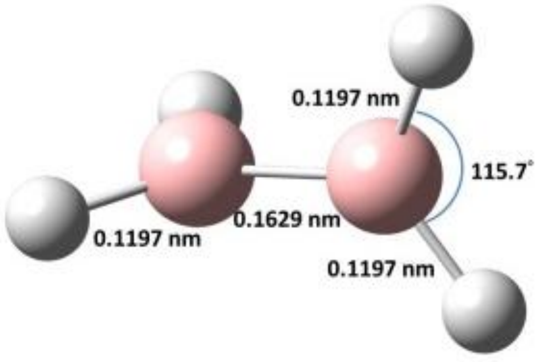

$^{11}B_2H_4$ (D_{2d})		$^{10}B_2H_4$ (D_{2d})		$^{11}B^{10}BH_4$ (C_{2v})	
mode (symmetry)	ν / cm^{-1} (int.)[a]	mode (symmetry)	ν / cm^{-1} (int.)[a]	mode (symmetry)	ν / cm^{-1} (int.)[a]
ν_1 (A_1)	2478.4 (0)	ν_1 (A_1)	2486.0 (0)	ν_1 (A_1)	2482.7(1.5)
ν_2 (A_1)	1153.6 (0)	ν_2 (A_1)	1172.2 (0)	ν_2 (A_1)	2460.6(62.3)
ν_3 (A_1)	838.5 (0)	ν_3 (A_1)	862.4 (0)	ν_3 (A_1)	1163.0(0)
ν_4 (B_1)	545.1 (0)	ν_4 (B_1)	545.2 (0)	ν_4 (A_1)	1096.2 (1.9)
ν_5 (B_2)	2458.2 (63.6)	ν_5 (B_2)	2463.9 (64.0)	ν_5 (A_1)	850.6 (0)
ν_6 (B_2)	1093.7 (1.9)	ν_6 (B_2)	1099.5 (1.9)	ν_6 (A_2)	545.1 (0)
ν_7 (E)	2519.4 (171.6)	ν_7 (E)	2533.0(172.0)	ν_7 (B_1)	2519.7 (85.6)
ν_8 (E)	958.7 (50.3)	ν_8 (E)	975.0 (51.9)	ν_8 (B_1)	972.2 (25.8)
ν_9 (E)	375.8 (6.3)	ν_9 (E)	377.8 (6.4)	ν_9 (B_1)	375.6 (3.1)
				ν_{10} (B_2)	2533.1 (86.0)
				ν_{11} (B_2)	961.4 (25.2)
				ν_{12} (B_2)	377.8 (3.2)

[a]Enthalpy of formation and vibrational wavenumbers, scaled by 0.967, calculated with program Gaussian 09, B3LYP method and basis set 6-311++G** (B3LYP/6-311++G**); calculated intensities have unit km mol^{-1}.

$^{11}B_2D_4$ (D_{2d})		$^{10}B_2D_4$ (D_{2d})		$^{11}B^{10}BD_4$ (C_{2v})	
mode (symmetry)	ν / cm^{-1} (int.)[a]	mode (symmetry)	ν / cm^{-1} (int.)[a]	mode (symmetry)	ν / cm^{-1} (int.)[a]
ν_1 (A_1)	1821.0 (0)	ν_1 (A_1)	1829.4 (0)	ν_1 (A_1)	1822.8 (0.7)
ν_2 (A_1)	979.9 (0)	ν_2 (A_1)	1007.1 (0)	ν_2 (A_1)	1783.2 (33.8)
ν_3 (A_1)	679.9 (0)	ν_3 (A_1)	682.8 (0)	ν_3 (A_1)	991.6 (0)
ν_4 (B_1)	387.2 (0)	ν_4 (B_1)	385.6 (0)	ν_4 (A_1)	815.3 (0.8)
ν_5 (B_2)	1787.5 (32.6)	ν_5 (B_2)	1788.1 (34.8)	ν_5 (A_1)	680.0 (0)
ν_6 (B_2)	816.9 (1.0)	ν_6 (B_2)	819.1 (0.8)	ν_6 (A_2)	385.6 (0)
ν_7 (E)	1883.4 (84.5)	ν_7 (E)	1894.6 (91.0)	ν_7 (B_1)	1876.4 (45.1)
ν_8 (E)	787.1 (33.3)	ν_8 (E)	804.1 (35.2)	ν_8 (B_1)	801.4 (17.5)
ν_9 (E)	282.1 (4.4)	ν_9 (E)	281.4 (3.7)	ν_9 (B_1)	279.1 (1.8)
				ν_{10} (B_2)	1894.8 (45.5)
				ν_{11} (B_2)	787.6 (16.9)
				ν_{12} (B_2)	281.2 (1.8)

[a]Enthalpy of formation and vibrational wavenumbers, scaled by 0.967, calculated with program Gaussian 09, B3LYP method and basis set 6-311++G** (B3LYP/6-311++G**); calculated intensities have unit km mol^{-1}.

6(g) B_2H_5 (C_{2v}), ΔH_f = 232.9 kJ mol^{-1}

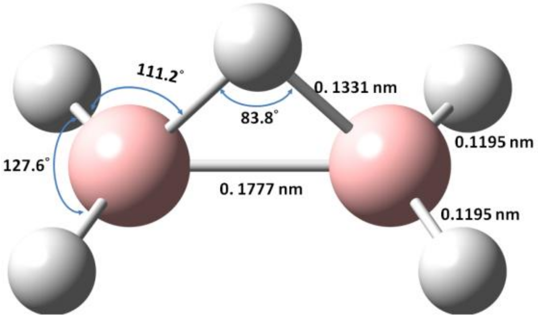

	$^{11}B_2H_5$	$^{10}B^{11}BH_5$	$^{11}B_2D_5$
mode (symmetry)	ν / cm^{-1} (int.)[a]	ν / cm^{-1} (int.)[a]	ν / cm^{-1} (int.)[a]
ν_1 (A_1)	2529.4 (4.3)	2532.3 (7.3)	1828.3 (4.0)
ν_2 (A_1)	1799.7 (9.5)	1803.1 (9.9)	1320.6 (6.9)
ν_3 (A_1)	1115.2 (8.6)	1118.6 (9.4)	851.5 (3.6)
ν_4 (A_1)	882.2 (3.8)	889.9 (3.7)	724.2 (2.6)
ν_5 (A_1)	640.7 (4.1)	650.1 (4.3)	525.5 (2.9)
ν_6 (A_2)	2633.7 (0)	2638.6 (19.3)	1976.3 (0)
ν_7 (A_2)	855.0 (0)	859.2 (0)	657.0 (0)
ν_8 (A_2)	151.4 (0)	151.7 (0)	110.8 (0)
ν_9 (B_1)	2651.6 (153.0)	2663.2 (134.9)	1992.1 (89.0)
ν_{10} (B_1)	960.7 (5.1)	962.0 (5.0)	695.7 (2.4)
ν_{11} (B_1)	420.7 (2.4)	420.7 (2.4)	297.9 (1.2)
ν_{12} (B_2)	2517.5 (82.4)	2519.2 (81.6)	1811.0 (63.4)
ν_{13} (B_2)	1656.4 (392.2)	1657.8 (395.0)	1192.4 (224.4)
ν_{14} (B_2)	1080.3 (103.1)	1083.7 (102.0)	810.4 (47.8)
ν_{15} (B_2)	766.3 (5.3)	772.2 (5.3)	622.0 (2.6)

[a]Enthalpy of formation and vibrational wavenumbers, scaled by 0.967, calculated with program Gaussian 09, B3LYP method and basis set 6-311++G** (B3LYP/6-311++G**); calculated intensities have unit km mol^{-1}.

6(h) B_2H_5 (C_s), ΔH_f = 259.8 kJ mol^{-1}

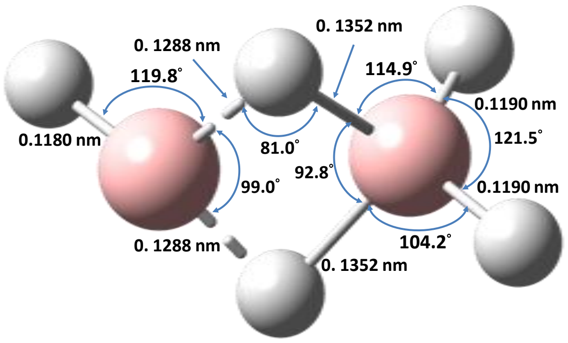

	$^{11}B_2H_5$	$^{10}B^{11}BH_5$	$^{11}B^{10}BH_5$	$^{11}B_2D_5$
mode (symmetry)	ν / cm^{-1} (int.)[a]	ν / cm^{-1} (int.)[a]	ν / cm^{-1} (int.)[a]	ν / cm^{-1} (int.)[a]
ν_1 (A')	2612.9 (69.1)	2624.5 (68.2)	2613.3 (79.5)	1936.0 (63.7)
ν_2 (A')	2584.6 (61.3)	2584.7 (64.4)	2599.2 (51.3)	1933.1 (13.2)
ν_3 (A')	2493.0 (71.4)	2493.0 (71.2)	2498.5 (74.2)	1803.8 (50.6)
ν_4 (A')	2139.4 (20.1)	2140.4 (20.2)	2139.5 (20)	1521.0 (10.7)
ν_5 (A')	1437.4 (212.8)	1439.9 (212.5)	1439.4 (215.9)	1047.7 (119.5)
ν_6 (A')	1133.8 (38.5)	1133.9 (38.2)	1139.7 (37.8)	864.8 (13.4)
ν_7 (A')	882.9 (1.5)	888.2 (1.1)	891.8 (1.5)	755.0 (0.9)
ν_8 (A')	836.4 (3.9)	848.6 (4.6)	850.6 (3.3)	685.4 (2.1)
ν_9 (A')	635.3 (0.2)	642.5 (0.3)	636.3 (0.3)	488.1 (0.5)
ν_{10} (A')	397.5 (20.3)	398.4 (20.4)	397.9 (20.4)	286.7 (10.8)
ν_{11} (A")	2035.3 (0.1)	2042.5 (0.1)	2037.0 (0.1)	1502.5 (0.1)
ν_{12} (A")	1590.3 (0.6)	1590.4 (0.6)	1593.8 (0.6)	1152.3 (0.8)
ν_{13} (A")	937.2 (11.1)	937.2 (11.1)	944.7 (11.3)	718.6 (6.4)
ν_{14} (A")	816.8 (0.2)	817.6 (0.2)	816.9 (0.3)	615.0 (0.3)
ν_{15} (A")	803.8 (0)	808.6 (0)	806.6 (0)	577.3 (0.1)

[a]Enthalpy of formation and vibrational wavenumbers, scaled by 0.967, calculated with program Gaussian 09, B3LYP method and basis set 6-311++G** (B3LYP/6-311++G**); calculated intensities have unit km mol^{-1}.

6(i) B_2H_6 (D_{2h}), ΔH_f = 50.1 kJ mol^{-1}

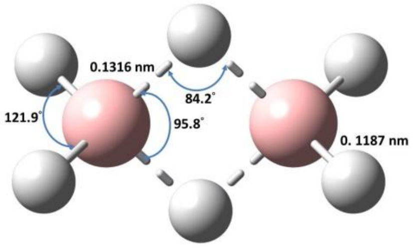

mode (symmetry)	$^{11}B_2H_6$ ν / cm^{-1} (int.)[a]	$^{10}B^{11}BH_6$ ν / cm^{-1} (int.)[a]	$^{11}B_2D_6$ ν / cm^{-1} (int.)[a]
ν_1 (A_g)	2524.2 (0)	2527.7 (6.1)	1833.1 (0)
ν_2 (A_g)	2094.9 (0)	2095.3 (0)	1487.4 (0)
ν_3 (A_g)	1160.0 (0)	1163.7 (7.1)	885.6 (0)
ν_4 (A_g)	770.6 (0)	786.4 (0)	692.9 (0)
ν_5 (A_u)	820.7 (0)	820.7 (0)	580.6 (0)
ν_6 (B_{1g})	2595.9 (0)	2600.3 (28.4)	1946.3 (0)
ν_7 (B_{1g})	905.3 (0)	910.5 (0)	721.9 (0)
ν_8 (B_{1u})	1915.0 (8.8)	1920.4 (9.0)	1430.4 (6.7)
ν_9 (B_{1u})	958.6 (16.6)	961.2 (16.6)	707.9 (8.6)
ν_{10} (B_{2g})	1784.8 (0)	1785.9 (0)	1280.6 (0)
ν_{11} (B_{2g})	863.6 (0)	870.8 (0)	707.2 (0)
ν_{12} (B_{2u})	2610.6 (185.5)	2621.6 (158.3)	1954.2 (104.1)
ν_{13} (B_{2u})	917.2 (0.2)	921.0 (0.2)	675.6 (0)
ν_{14} (B_{2u})	346.6 (16.4)	346.6 (16.4)	245.3 (8.2)
ν_{15} (B_{3g})	989.6 (0)	989.6 (0)	700.0 (0)
ν_{16} (B_{3u})	2511.0 (148.8)	2513.0 (146.1)	1813.2 (113.9)
ν_{17} (B_{3u})	1645.6 (472.0)	1648.1 (475.5)	1199.8 (266.6)
ν_{18} (B_{3u})	1151.2 (76.7)	1153.6 (68.3)	853.2 (26.1)

[a]Enthalpy of formation and vibrational wavenumbers, scaled by 0.967, calculated with program Gaussian 09, B3LYP method and basis set 6-311++G** (B3LYP/6-311++G**); calculated intensities have unit km mol^{-1}.

7. Wavenumber/cm^{-1} and intensity/km mol^{-1} of calculated fundamental vibrational modes for various isotopic $B_2H_4^+$ (C_{2v}) and $B_2D_4^+$ (C_{2v})

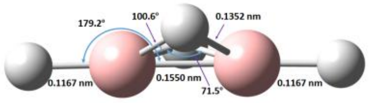

Table S7. Wavenumber/cm^{-1} and intensity/km mol^{-1} of calculated fundamental vibrational modes for various isotopic $B_2H_4^+$ (C_{2v}) and $B_2D_4^+$ (C_{2v})

	$^{11}B_2H_4^+$		$^{11}B^{10}BH_4^+$		$^{10}B_2H_4^+$	
mode(sym)	calc. cm^{-1} (int.)[a]	calc. cm^{-1} [b]	calc. cm^{-1} (int.)[a]	calc. cm^{-1} [b]	calc. cm^{-1} (int.)[a]	calc. cm^{-1} [b]
ν_1 (A_1)	2773.8 (0)	2767.2	2784.3 (0.91)	2776.6	2784.3 (0.91)	2785.6
ν_2 (A_1)	2050.7 (4.45)	2000.3	2051.2 (4.37)	2002.3	2051.2 (4.37)	2013.6
ν_3 (A_1)	1112.8 (0.35)	1130.2	1135.6 (0.42)	1134.9	1158.2 (0.49)	1156.3
ν_4 (A_1)	739.6 (6.41)	658.6	742.1 (5.86)	660.8	744.5 (5.35)	662.9
ν_5 (A_1)	540.7 (9.77)	558.8	541.2 (10.16)	551.8	541.6 (10.49)	551.8
ν_6 (A_2)	1545.2 (0)	1475.4	1547.0 (0.01)	1478.1	1548.8 (0)	1480.7
ν_7 (A_2)	687.0 (0)	699.8	693.2 (0.01)	706.0	699.6 (0)	712.4
ν_8 (B_1)	2098.1 (16.79)	2165.2	2102.7 (16.12)	2171.5	2107.4 (15.47)	2178.2
ν_9 (B_1)	758.4 (5.72)	782.7	759.6 (6.09)	783.9	760.7 (6.43)	784.9
ν_{10} (B_2)	2744.1 (29.18)	2731.6	2748.6 (26.91)	2736.9	2756.1 (26.46)	2743.0
ν_{11} (B_2)	1398.9 (124.19)	1315.4	1401.5 (126.55)	1317.8	1403.7 (128.78)	1319.7
ν_{12} (B_2)	489.2 (2.76)	639.8	493.5 (2.86)	646.2	498.0 (2.94)	653.1
mode(sym.)	$^{11}B_2D_4^+$		$^{11}B^{10}BD_4^+$		$^{10}B_2D_4^+$	
ν_1 (A_1)	calc. cm^{-1} (int.)[a]	calc. cm^{-1} [b]	calc. cm^{-1} (int.)[a]	calc. cm^{-1} [b]	calc. cm^{-1} (int.)[a]	calc. cm^{-1} [b]
ν_2 (A_1)	2103.8 (0.07)	2136.4	2121.0 (0.09)	2161.8	2135.3 (0.11)	2162.0
ν_3 (A_1)	1457.8 (1.45)	1436.9	1458.6 (1.38)	1439.6	1459.3 (1.30)	1441.5
ν_4 (A_1)	1031.2 (0.77)	1045.3	1049.5 (6.54)	1067.3	1064.3 (0.86)	1067.6
ν_5 (A_1)	556.0 (0.10)	518.6	559.3 (0.03)	521.9	562.3 (0)	524.6
ν_6 (A_2)	387.3 (8.18)	394.1	387.6 (8.37)	394.6	388.0 (8.74)	394.5
ν_7 (A_2)	1120.5 (0)	1061.9	1123.4 (0.01)	1067.8	1126.5 (0)	1070.4
ν_8 (B_1)	565.2 (0)	577.6	572.3 (0.31)	585.0	578.6 (0)	591.3
ν_9 (B_1)	1549.3 (3.14)	1488.5	1555.7 (2.77)	1495.2	1562.0 (2.43)	1503.3
ν_{10} (B_2)	550.8 (6.77)	568.3	551.6 (6.90)	569.1	553.3 (7.60)	570.8
ν_{11} (B_2)	2026.3 (4.21)	2035.0	2033.3 (3.59)	2042.0	2043.2 (2.93)	2051.1
ν_{12} (B_2)	1020.2 (84.0)	985.3	1021.3 (80.3)	988.7	1025.5 (88.0)	990.8

[a] These wavenumbers/cm^{-1}, scaled by 0.967, and intensities/km mol^{-1} within parentheses were calculated in an harmonic approximation.

[b] These wavenumbers/cm^{-1} were calculated in an anharmonic approximation.

8. Wavenumber/cm^{-1} and intensity/km mol^{-1} of calculated fundamental vibrational modes and NIST data for $^{11}B_2H_6$

Table S8. Wavenumber/cm^{-1} and intensity/km mol^{-1} of calculated fundamental vibrational modes and NIST data for $^{11}B_2H_6$

mode (symmetry)	type of mode	data from NIST[a]	calc. ν (int.)[b]
$\nu_1(A_g)$	BH_2 s-str	2524	2524.2 (0)
$\nu_2(A_g)$	ring str	2104	2094.9 (0)
$\nu_3(A_g)$	BH_2 sciss	1180	1160.0 (0)
$\nu_4(A_g)$	ring deform	794	770.6 (0)
$\nu_5(A_u)$	BH_2 twist	833	820.7 (0)
$\nu_{10}(B_{1g})$	ring str	1768	1784.8 (0)
$\nu_{11}(B_{1g})$	BH_2 wag	850	863.6 (0)
$\nu_{12}(B_{1u})$	BH_2 a-str	2612	2610.6 (185.5)
$\nu_{13}(B_{1u})$	BH_2 rock	950	917.2 (0.2)
$\nu_{14}(B_{1u})$	ring pucker	368	346.6 (16.4)
$\nu_6(B_{2g})$	BH_2 a-str	2591	2595.9 (0)
$\nu_7(B_{2g})$	BH_2 rock	915	905.3 (0)
$\nu_8(B_{2u})$	ring str	1915	1915.0 (8.8)
$\nu_9(B_{2u})$	BH_2 wag	973	958.6 (16.6)
$\nu_{15}(B_{3g})$	BH_2 twist	1012	989.6 (0)
$\nu_{16}(B_{3u})$	BH_2 s-str	2525	2511.0 (148.8)
$\nu_{17}(B_{3u})$	ring deform	1602	1645.6 (472.0)
$\nu_{18}(B_{3u})$	BH_2 sciss	1177	1151.2 (76.7)

[a] data from NIST Chemistry WebBook at http://webbook.nist.gov/chemistry/

[b]Vibrational wavenumbers (unit cm^{-1}), scaled by 0.967, calculated with program Gaussian 09, B3LYP method and basis set 6-311++G** (B3LYP/6-311++G**); calculated intensities have unit km mol^{-1}.
